# Vps34 puts the ‘e’ in eTreg cells

**DOI:** 10.1371/journal.pbio.3003109

**Published:** 2025-04-14

**Authors:** Ruchi Saxena, You-Wen He

**Affiliations:** Department of Integrative Immunobiology, Duke University School of Medicine, Durham, North Carolina, United States of America

## Abstract

Regulatory T cells (Tregs) exist in distinct subsets, but key regulators of their heterogeneity remain unclear. A recent PLOS Biology study shows that the class III PI3K Vps34 acts as a master orchestrator, driving effector Treg generation and maintenance.

Regulatory T cells (Tregs) are essential gatekeepers of immune tolerance, existing as heterogeneous subsets in distinct functional states [1, 2]. Their phenotypic heterogeneity reflects the need for context-dependent immune suppression and adaptation to the homeostatic environment. Among the heterogeneous Tregs, central Tregs (cTregs) and effector Tregs (eTregs) play critical roles in immune regulation [1, 2]. However, the mechanisms governing their differentiation and maintenance remain incompletely understood. A recent PLOS Biology study by Norton and colleagues [3] conducted an extensive genetic and multi-omics investigation on the role of class III phosphatidylinositol-3-kinase vacuolar protein sorting 34 (Vps34) in Treg differentiation and function. Vps34 plays critical roles in autophagy, endocytic trafficking, and endocytosis and is essential for the maintenance and function of peripheral Tregs, as demonstrated in a pan-T cell deletion model [4]. Given the broad impact of Vps34 deletion on conventional T cell compartments [4, 5], it was necessary to investigate its function in Tregs specifically.

Here [3], the authors demonstrated that Treg-specific deletion of Vps34 disrupts the generation of terminal eTregs during perinatal life and their maintenance and function post-perinatally. In concordance with findings from two recent publications [6, 7], this firmly establishes Vps34 as a master orchestrator of terminal eTreg generation and maintenance perinatally and post-perinatally (Fig 1).

**Fig 1:**
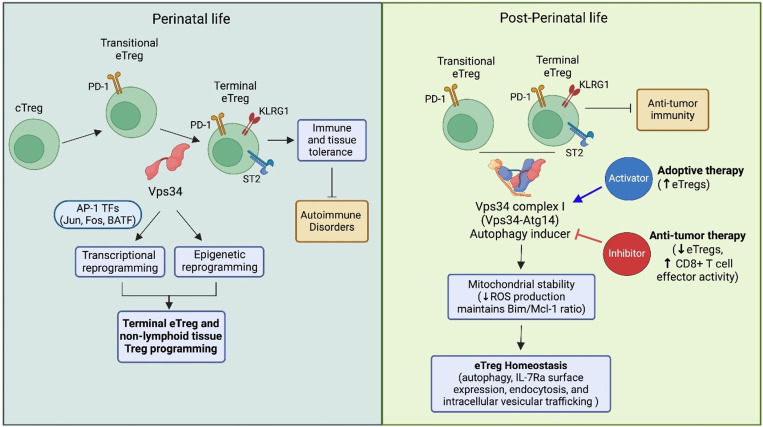
Regulation of Treg differentiation and function by Vps34 perinatally and post-perinatally and its clinical implications. Vps34 plays a crucial role in the differentiation of conventional Tregs (cTregs) into transitional effector Tregs (eTregs; PD‐1+) and subsequently into terminal effector Tregs (KLRG1^+^, ST2^+^) during perinatal development and their maintenance and function through post-perinatal life. Vps34, through AP‐1 transcription factors (TFs; Jun, Fos, BATF), facilitates transcriptional and epigenetic reprogramming essential for immune tolerance, thereby averting autoimmunity during perinatal life. Post-perinatally, the Vps34–Atg14 complex promotes autophagy, stabilizes mitochondria (reducing reactive oxygen species and regulating the Bim/Mcl-1 ratio), and sustains eTreg homeostasis (e.g., IL-7Rα/CD127 expression). Modulating Vps34 complex pathways—either by activation or inhibition—holds potential for clinical applications in treating autoimmune diseases and cancer. The authors acknowledge Biorender for figure generation. AP-1, activator protein 1; Atg14, autophagy‐related gene 14; BATF, basic leucine zipper ATF‐like transcription factor; Bim, Bcl‐2-interacting mediator of cell death; IL‐7Rα, interleukin‐7 receptor alpha chain (CD127); KLRG1, killer cell lectin‐like receptor G1; Mcl‐1, myeloid cell leukemia 1; PD‐1, programmed cell death protein 1; ROS, reactive oxygen species; ST2, suppression of tumorigenicity 2; Vps34, vacuolar protein sorting 34.

Norton and colleagues generated a Treg-specific Vps34 knockout model and observed that the mice rapidly developed a Type I-dominant, Scurfy-like systemic autoimmune disorder [3]. Scurfy mice (with scaly and flaky skin due to dermatitis) have defective Tregs with Foxp3 mutation. Vps34 deletion impaired the differentiation of terminal KLRG1⁺Nfil3⁺ST2⁺ eTregs during perinatal life and their homeostasis post-perinatally in a cell-intrinsic manner (Fig 1). The Scurfy-like phenotype, along with a reduction in eTregs or mature Tregs, was also observed in two independent studies using a similar Treg-specific Vps34 deletion approach [6, 7]. Further multi-omics analysis by Norton and colleagues revealed two major transcriptional and epigenetic alterations in Vps34-deficient Tregs. First, these cells exhibited an increased KLRG1^−^Nfil3⁺ transitional Treg signature and a reduction in the KLRG1⁺Nfil3⁺ terminal Treg signature. Second, they displayed enhanced epigenetic signatures associated with transitional Tregs but reduced terminal and non-lymphoid tissue-associated epigenetic programs. Collectively, these findings suggest that Vps34 orchestrates terminal and non-lymphoid tissue Treg-associated transcriptional and epigenetic reprogramming [3] (Fig 1).

The reduced presence of terminal eTregs (or mature Tregs) in the absence of Vps34 is partly attributed to increased apoptosis, a consistent observation across three independent studies [3,6,7]. This heightened cell death is associated with mitochondrial abnormalities, elevated reactive oxygen species (ROS), and increased Bim/Mcl-1 and Bim/Bcl-2 ratios. An increase in the ratio of the proapoptotic protein Bim to the antiapoptotic proteins Mcl-2 and Bcl-2 results in activation of the intrinsic apoptotic death pathway. The survival defect is partially linked to Vps34’s role in autophagy, a critical intracellular process required for mitochondrial clearance in T lymphocytes [8] (Fig 1). Supporting this, Norton and colleagues examined Treg development and function in mice with lineage-specific deletion of Atg14 or Uvrag, two Vps34-interacting proteins that form Complex I (initiating autophagy) and Complex II (facilitating endosome maturation and trafficking to the lysosome), respectively. Deletion of Atg14, but not Uvrag, in Tregs partially recapitulated the phenotypes observed in Vps34-deficient mice, including mitochondrial abnormalities, elevated ROS, increased Bim/Mcl-1 ratio, reduced eTreg numbers, and impaired non-lymphoid Treg accumulation and function after perinatal life [3] (Fig 1). However, unlike Vps34-deficient Tregs, Atg14-deficient Tregs did not induce a fatal, early-onset autoimmune disorder. Similarly, Tregs with Atg7 deletion, another key autophagy-related gene, only partially mimicked the defects seen in Vps34-deficient Tregs [6]. These findings suggest that the function of Vps34 in orchestrating eTregs existence extends beyond autophagy and likely regulates eTreg homeostasis through additional pathways, including IL-7Rα surface expression, endocytosis, and intracellular vesicular trafficking [5,7] (Fig 1). It was previously shown that Vps34-deficient T cells exhibit increased cell death and reduced IL-7Rαsurface expression due to impaired intracellular trafficking of IL-7Rα through the retromer pathway for surface display [5].

The findings of Norton and colleagues provide novel insights into the role of Vps34 in orchestrating eTregs and have significant clinical implications. One potential therapeutic avenue is the activation of Vps34 in Tregs to generate highly potent eTregs in vivo or *ex vivo* for cellular therapy in autoimmune diseases, as eTregs exhibit greater immunosuppressive function than cTregs [1,2]. Clinical trials of Treg-based therapies have demonstrated a good safety profile and some clinical benefits [9]. However, Tregs from patients with autoimmune diseases are often functionally impaired, posing challenges for ex vivo expansion. Activating Vps34 through its signaling complexes may enhance the production of highly potent eTregs for adoptive therapy (Fig 1). Conversely, both Norton and colleagues and Feng and colleagues showed that mice with Vps34-deficient Tregs have enhanced antitumor immunity [3,7], suggesting that pharmaceutical inhibition of Vps34 could improve cancer treatment. Notably, Vps34 inhibition not only disrupts the immunosuppressive function of eTregs but also enhances the effector activity of antitumor-specific CD8⁺ T cells. A recent study found that Vps34-dependent autophagy in activated CD8⁺ T cells degrades key effector molecules, including perforin and granzymes [10]. Thus, inhibiting Vps34 prevents this degradation, potentially enhancing antitumor responses through both blockade of Tregs and enhanced killing by effector T cells (Fig 1).

In summary, Vps34 has emerged as a key regulator of eTreg differentiation, maintenance, and function. Norton and colleagues and other recent studies have demonstrated that Treg-specific deletion of Vps34 leads to a loss of terminal eTregs, increased apoptosis, mitochondrial dysfunction, and a fatal autoimmune disorder. Mechanistically, Vps34 may regulate eTreg homeostasis through both autophagy-dependent and autophagy-independent pathways, including IL-7Rα expression, endocytosis, and intracellular trafficking. The clinical implications of these findings are significant, as activating Vps34 could enhance the production of potent eTregs for autoimmune therapy while its inhibition may improve antitumor immunity by disrupting Treg-mediated suppression and enhancing CD8⁺ T cell effector functions.

While these studies have established Vps34 as a central regulator of eTregs, several key questions remain. Future research should explore how Vps34 integrates with other signaling pathways that govern Treg plasticity and function in different tissue microenvironments. Additionally, identifying pharmacological modulators of Vps34 that selectively enhance or inhibit its different aspects of the function in Tregs could open new avenues for therapeutic interventions in autoimmune diseases and cancer. Further investigation into the autophagy-independent functions of Vps34, particularly its role in intracellular trafficking and cytokine receptor regulation, will be critical to fully understanding its impact on immune homeostasis. Finally, clinical studies testing the effects of Vps34 activation or inhibition in human Tregs will be essential to translating these findings into viable immunotherapies.

## Abbreviations

cTregs: central Tregs
eTregs: effector Tregs
ROS: reactive oxygen species
TFs: transcription factors
Tregs: regulatory T cells
Vps34: vacuolar protein sorting 34
